# Prospective evaluation of spinal cord stimulation in refractory angina: insights from a single-center cohort

**DOI:** 10.3389/fcvm.2025.1681623

**Published:** 2025-12-08

**Authors:** Nigar Z. Gasimova, Artem A. Paltsev, Nikita A. Zayachkovskiy, Viktoriya G. Nezdorovina, Oleg V. Nezdorovin, Mariya A. Bortsova, Mariya A. Osipova, Nikita S. Remizov, Heber Ivan Condori Leandro, Aleksandra S. Vatian, Dmitry S. Lebedev, Evgeny N. Mikhaylov

**Affiliations:** 1 Almazov National Medical Research Center, Saint-Petersburg, Russia; 2 ITMO University, Saint-Petersburg, Russia

**Keywords:** coronary artery disease, refractory angina, neuromodulation, spinal cord stimulation, efficacy, safety, nitrate use, decision tree

## Abstract

**Background:**

Refractory angina pectoris represents a significant clinical challenge characterized by persistent chest pain despite maximally tolerated medical therapy and optimal revascularization attempts. This study evaluates the efficacy and safety of invasive spinal cord stimulation (SCS) in patients with refractory angina.

**Methods:**

Twenty-one patients underwent neurostimulation system implantation between 2022 and 2023 (mean age 62.8 ± 7, 12 males) diagnosed with refractory angina. All patients received both chronic continuous stimulation and on-demand stimulation to alleviate anginal pain episodes. The primary endpoint was changes in the Seattle Angina Questionnaire (SAQ-19) scores. Secondary endpoints included changes in the Short Form Health Survey (SF-36), incidence of major adverse cardiovascular events, device-related complications, and nitrate use.

**Results:**

Eighteen patients completed the final follow-up. No statistically significant improvements were observed in any SAQ-19 domains. However, reductions in pain scores (41% vs. 53%, *p* = 0.007) and improvements in mental health scores (61% vs. 72%, *p* = 0.021) and physical functionating (36 vs. 52%, *p* = 0.017) were noted on the SF-36. Device extraction occurred in three cases (14%)—two due to pocket infection and one at the patient's request. Four lead repositioning procedures were performed (19%). Hospitalization rates significantly decreased over the one-year observation period, from 3.8 to 0.5 hospitalizations per patient (*p* = 0.03). Recurrent acute coronary syndrome was noted in one patient, requiring additional coronary stentings. Notably, although no significant reduction in nitrate therapy was achieved, the treatment regimen prevented any new nitrate prescriptions during the study period.

**Conclusion:**

SCS did not significantly improve the primary endpoint of disease-specific quality of life (SAQ-19). However, treatment was associated with secondary benefits, including improvements in physical functionating, pain and mental health (SF-36) and a significant reduction in hospitalization rates. Complication rates were significant.

## 1 Introduction

Refractory angina is defined as a chronic condition persisting for at least six months, caused by myocardial ischemia triggered by obstructive coronary artery disease (CAD) and/or other mechanisms, represents a significant clinical challenge. It is characterized by persistent angina or angina-equivalent symptoms despite maximally tolerated stratified anti-ischemic therapy and achievable indicated revascularization (1). The pathophysiological mechanisms of refractory angina can affect any level of coronary circulation and are not limited to obstructive artery disease. The supply-demand imbalance is often exacerbated by concurrent microvascular dysfunction or vasospasm. The underlying ischemic-neural mechanism involves the sensitization of cardiac pain pathways due to chronic myocardial ischemia, which triggers aberrant pain signals to the spinal cord and brain. Over time, this process leads to central sensitization, heightening the sensitivity of pain pathways and amplifying pain perception even with minimal ischemia. Concurrently, dysregulation of the autonomic nervous system (ANS) further complicates the condition by impairing cardiac regulation. As a result, the persistent pain becomes unresponsive to standard revascularization and medical therapies (2). Therefore, refractory angina is driven by persistent myocardial ischemia owing to a supply-demand mismatch, compounded by endothelial dysfunction and heightened neural sensitization: myocardial ischemia triggers local release of metabolic byproducts (lactate, adenosine), activating nociceptive afferents; endothelial dysfunction reduces flow-mediated dilation, promotes microvascular spasm, and impairs adaptive responses to increased oxygen demand; chronic pain signaling in cardiac neurons may result in exaggerated pain responses. This chronic condition affects a growing number of patients, fueled by enhanced diagnostic precision and the increasing recognition of complex coronary pathologies such as combined atherosclerosis and vasospasm. Refractory angina affects an estimated 5%–10% of patients with stable CAD. This translates to a substantial annual incidence of 50,000–100,000 new cases in the United States and 30,000–50,000 in Europe (3). Standard pharmacological regimens often require multi-drug approaches, yet many patients continue to experience debilitating symptoms and frequent hospitalizations, underscoring the urgent need for alternative therapeutic strategies.

Invasive spinal cord stimulation (SCS) has emerged as a promising neuromodulation technique (4). The neurophysiological mechanism of SCS involves its action on inhibitory interneurons within the substantia gelatinosa. This process leads to a reduction in sympathetic afferent outflow and the release of inhibitory neurotransmitters, such as GABA, serotonin, and enkephalins, ultimately suppressing both nociceptive signaling and cardiac sympathetic tone. SCS does not block the pain itself. Instead, it uses a non-painful “tingling” sensation (paresthesia) to modulate the nervous system's processing of pain. It achieves this through two primary, interconnected mechanisms: 1) Aβ fiber-mediated gating: the electrical stimulation selectively activates large-diameter Aβ sensory fibers, which “close the gate” on the pain signals carried by small-diameter Aδ and C fibers from the heart; 2) ANS modulation: the stimulation influences the sympathetic and parasympathetic branches of the ANS, leading to reduced cardiac workload, improved coronary blood flow, and a direct anti-ischemic effect (2).

However, due to procedural invasiveness, risk of complications, and heterogeneity in stimulation protocols, the role of SCS remains unclear and is absent from current clinical guidelines. Moreover, prior studies have reported mixed results, with inconsistent improvements in patient-reported outcomes and variable safety profiles (5–7).

We believe this shift is the result from several factors: the invasive nature of the therapy and associated complications, which may tilt the risk-benefit balance unfavorably—particularly because both initial implantation and electrode repositioning require interruption of antiplatelet therapy in high thrombotic risk patients; the lack of standardized stimulation protocols complicating comparisons across studies; and the impossibility of sham controls, as patients invariably perceive stimulation and can replicate it using remote devices.

Given these uncertainties, the aim of our study was to evaluate whether SCS reduces anginal pain, improves quality of life, and decreases hospitalizations in a clinically diverse cohort of refractory angina patients. Additionally, the complication rate was assessed in this real practice cohort.

## 2 Methods

### 2.1 Design

This single-center, single-arm prospective observational study was conducted between 2022 and 2024. It enrolled 21 patients with Canadian Cardiovascular Society (CCS) class III–IV refractory angina, who had persistent symptoms despite ≥6 months of guideline-directed optimal medical therapy and were deemed unsuitable for complete revascularization. Enrollment prioritized patients with advanced angina (CCS Class III–IV) and frequent hospitalizations (>3/year) due to cardiovascular conditions. The study cohort was identified from the patient pools of the specialized research laboratories. Inclusion criteria required clinical stability for at least 30 days prior to enrollment, signed informed consent, and demonstrated ability to manage the neurostimulator device, including charging and operating the programmer. Exclusion criteria included acute medical conditions such as myocardial infarction within the prior 30 days, stroke or transient ischemic attack, severe spinal trauma or contraindications to lumbar puncture, and a life expectancy of less than one year.

All patients underwent a standard clinical evaluation consistent with the diagnostic workup for CAD, including stress testing and coronary angiography. In cases with obstructive CAD, the inability to perform further revascularization and failure of pharmacological treatments were confirmed by a multidisciplinary team comprising a general cardiologist, invasive cardiologist, and cardiovascular surgeon.

Since ergonovine and acetylcholine provocation tests are not approved for clinical use in our country, the diagnosis of vasospastic angina was established based on clinical history, ECG evidence of ST-segment deviations, and documented coronary vasospasm during angiography in patients without hemodynamically significant coronary artery stenoses.

### 2.2 Electrode implantation procedures

Electrode implantation was performed in an x-ray equipped operating room with the patient in the prone position. Local anesthesia was administered to the skin and tissues at lumbar or thoracic levels (between vertebrae Th11-Th12, L3-L4, or L2-L3). The epidural space was accessed using a standard puncture technique under fluoroscopic guidance.

The first cylindrical 8-contact electrode (Linear 3–6, Boston Scientific, USA) was inserted slightly left of midline and positioned to cover spinal cord segments Th1 to Th5. A second stimulating electrode was inserted through the same puncture site, positioned symmetrically slightly to the right of midline. The upper thoracic spinal cord (Th1-Th5) is the target for SCS, because it serves as the central convergence point for nociceptive signals originating from the heart.

No initial test stimulation period with temporary (trial) electrodes was performed prior to permanent system implantation. The researchers opted for this protocol due to the following considerations: data from short-term trials may artificially inflate efficacy (8), extended hospitalization associated with test stimulation and its inherent procedural risks (9), coupled with its limited predictive value for sustained clinical efficacy (10).

Bilateral bipolar test stimulation of the electrodes at the Th1–Th5 level was conducted intraoperatively using a lead tester, adjusting stimulation parameters to the minimum perceptible level for the patient (stimulation frequency: 80 Hz; amplitude individualized based on paresthesia perception). A frequency of 80 Hz falls within the high-frequency stimulation range, which has been shown to modulate sympathetic activity and suppress the transmission of pain signals at the spinal level, possibly through the activation of descending inhibitory pathways and mechanisms related to Diffuse Noxious Inhibitory Controls (11).

**Figure 1:**
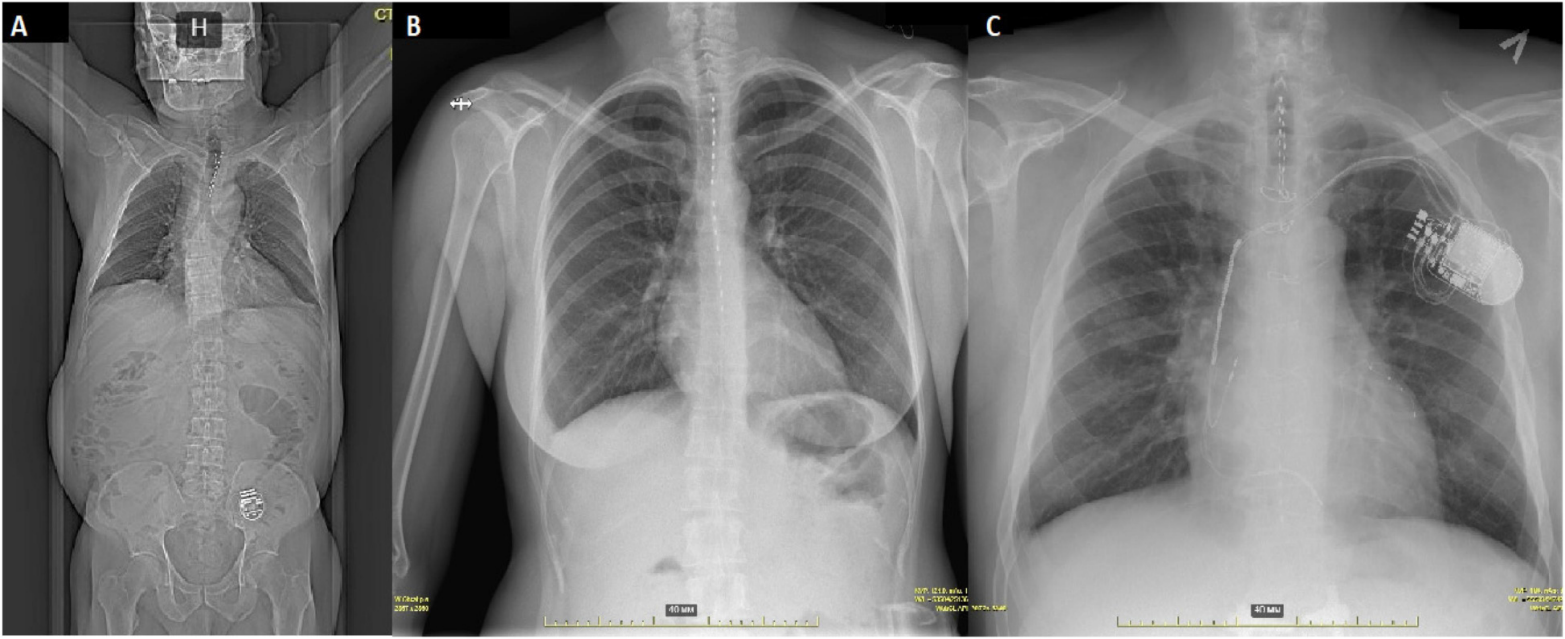
Radiographic imaging in different clinical cases. **(A)** Computed tomography showing normal electrode and pulse generator positioning. **(B)** Sequential electrode placement in a patient with generalized artery spasms, including coronary and celiac artery. **(C)** Spinal cord stimulation in a patient with a CRT-D device and an implanted stimulator for persistent spinal pain syndrome type 2.

Following successful electrode placement, skin incisions (3 cm) were made at implantation sites to secure electrodes to the fascia with sutures. A separate 5–7 cm lateral torso incision was made to create a subfascial pocket for the neurostimulator. Electrodes were tunneled subcutaneously to this pocket and connected to the neurostimulator device (Precision Montage, Boston Scientific, USA), which was implanted and the wounds closed in layers (Figures 1A,B).

Patients with pre-existing cardiac rhythm management devices underwent simultaneous interrogation of both the spinal cord stimulator and cardiac device to assess potential electromagnetic interference, including shock channel disturbance and pacemaker inhibition (Figure 1C).

Antithrombotic therapy was interrupted 2–5 days before the procedure according to current guidelines governing non-cardiac interventions.

### 2.3 Device programming and stimulation parameters

Stimulation parameters were initially adjusted intraoperatively and further optimized over the subsequent 1–5 days. Programming was performed using a dedicated analyzer and programming head.

The stimulation threshold was defined as the current intensity (measured in milliamperes) at which the patient first perceived stimulation, typically as paresthesia within dermatomes correlating to angina symptoms, adjusted individually for each patient.

Two stimulation modes were programmed: (1) a continuous chronic stimulation mode set to 85% of the threshold intensity, operating 24 h a day, 7 days a week; (2) an “angina relief” mode, in which patients experience typical stimulation-induced paresthesia in the dermatome corresponding to angina pectoris, activated on demand by the patient during episodes of anginal pain. The intensity, set at 85% of the individual paresthesia threshold, was chosen to ensure strong afferent stimulation of Aβ fibers without causing discomfort or muscle twitching. This approach aims to maximally engage non-painful “gate-closing” mechanisms in the spinal cord while minimizing the activation of nociceptive pathways (10, 12). All patients received thorough training on switching between modes using a handheld programmer and on recharging the pulse generator.

### 2.4 Follow up

Follow-up assessments were conducted at baseline, and then at 1, 6, and 12 months after hospital discharge. These assessments included physical examinations, completion of the Seattle Angina Questionnaire (SAQ-19) covering five domains: Physical Limitation, Angina Stability, Angina Frequency, Treatment Satisfaction, and Quality of Life; and the Short Form Health Survey (SF-36) covering eight domains: Physical Functioning, Role Physical, Pain, General Health, Vitality, Social Functioning, Role Emotional, and Mental Health. Additionally, echocardiography and stress echocardiography evaluations were performed at these time points. Collected medical history included documentation of all hospitalizations, cardiovascular events, and device-related complications (such as lead migration, lead fracture, and infection). Device-related adverse events were assessed at each scheduled visit via radiographic imaging and additionally on an unscheduled basis whenever symptoms of complications occurred (stimulation migration, local pain, fever).

In patients with cardiac implantable electronic devices, any adjustments to the neurostimulator's configuration and parameters were performed concurrently with device interrogation during follow-up visits.

### 2.5 Endpoints

The primary endpoint was the change in SAQ-19 scores, calculated as the mean across all five domains at baseline and at 12 months follow-up. Secondary endpoints included changes in SF-36 scores between baseline and 12 months, incidence of acute coronary syndrome, revascularization procedures, hospitalizations, nitrate usage, and device-related safety outcomes.

### 2.6 Statistical analysis

An initial exploratory statistical analysis was performed using a specialized software developed by the Research Center for Artificial Intelligence “Strong Artificial Intelligence in Industry” at ITMO University, Saint Petersburg. This tool was employed as part of an R&D initiative aimed at customizing and validating innovative AI-based solutions to enhance the productivity of medical and healthcare scientists.

Normality was assessed using the Shapiro–Wilk test. Continuous variables with approximately normal distribution are presented as mean ± standard deviation (SD). Categorical variables are expressed as counts and percentages. Comparisons of categorical variables between groups were performed using the chi-square test or Fisher's exact test, as appropriate. Data from the SAQ-19 and SF-36 questionnaires were analyzed using the paired Student's *t*-test for normally distributed variables and the Wilcoxon signed-rank test for non-normally distributed variables. Statistical significance was set at a two-tailed *p*-value <0.05, with Bonferroni correction applied as needed.

Sample size calculation was based on the assumption that the SAQ-19 “Treatment Satisfaction” domain would increase from an average of 50% among refractory angina patients to 80% after SCS system implantation, with a statistical power of 80% and a type I error probability of 0.05. According to these parameters, a minimum of 19 patients undergoing SCS would be required.

All conventional statistical analyses were performed using IBM SPSS Statistics version 26 (IBM Corp., Armonk, NY, USA) and Jamovi Software [2025, *jamovi* (Version 2.6) (Computer Software). Retrieved from https://www.jamovi.org].

### 2.7 Adversarial validation

To enhance the robustness of our findings, we applied *adversarial validation*, a machine learning technique used to detect distributional differences between data subsets. A Decision Tree classifier (Gini impurity criterion; IBM SPSS Statistics v26) was trained to identify systematic feature patterns distinguishing temporal subgroups within the cohort. Feature importance values derived from the model quantified each variable's relative contribution to classification performance, revealing which features most strongly influenced subgroup discrimination. Features with high importance scores indicated potential sources of internal variability within the cohort. By systematically quantifying these sources, this machine learning–based approach strengthened methodological rigor and consistency beyond what traditional statistical tests typically provide.

## 3 Results

### 3.1 Study population

**Figure 2:**
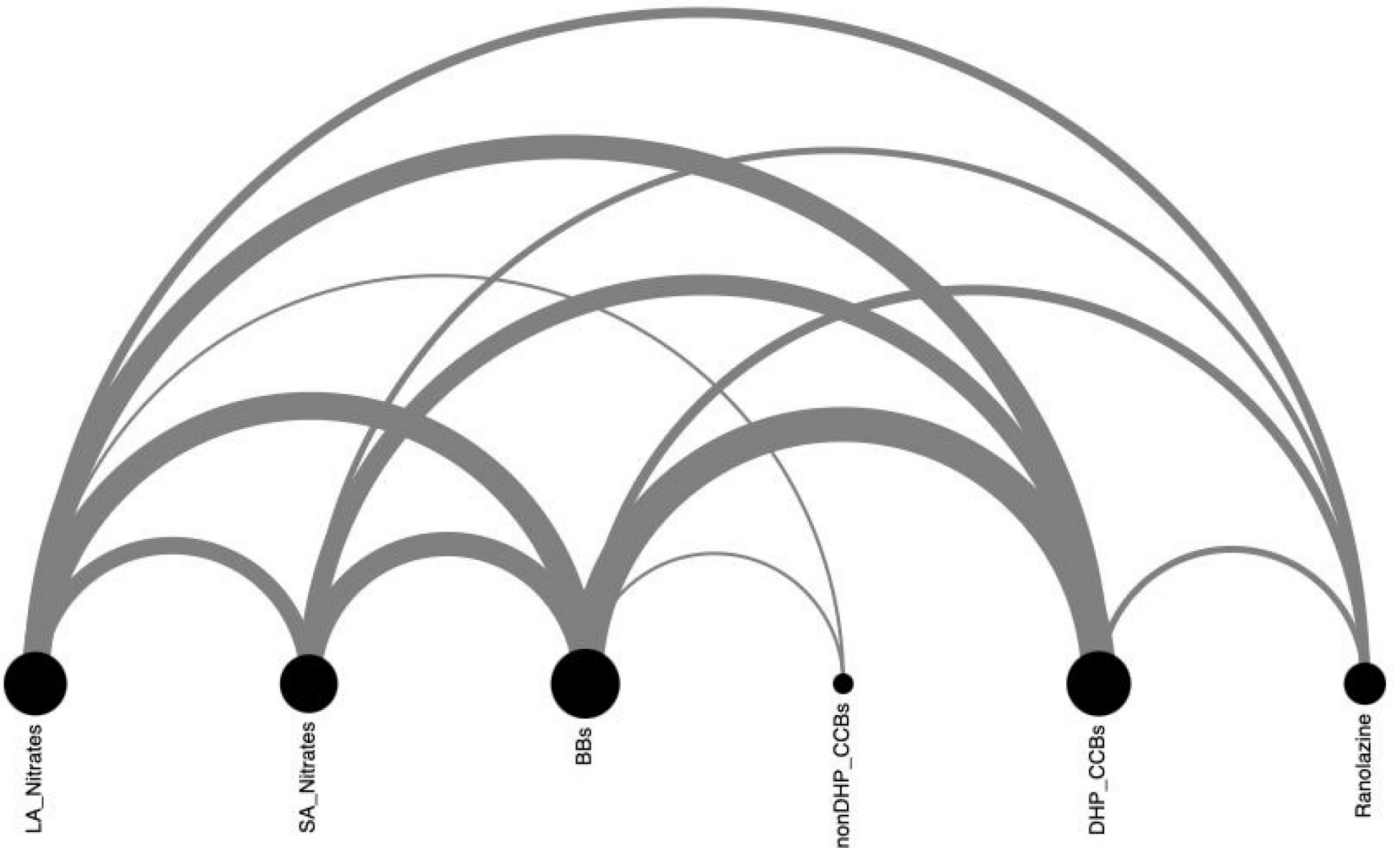
Pharmacological treatment of angina. DHP_CCBs, dihydropyridine calcium channel blocker; LA, long-acting; SA, short-acting; nonDHP_CCBs, non- dihydropyridine calcium channel blocker.

Twenty-one patients (57% male, mean age 62.8 ± 7 years) with refractory angina, preserved left ventricular function, and common comorbidities including hypertension and overweight status were enrolled. All participants had exhausted guideline-directed medical therapy and were unsuitable for complete revascularization. Most patients received complex pharmacological regimens, frequently combining beta-blockers with dihydropyridine calcium channel blockers or long-acting nitrates. Baseline characteristics and medication patterns are summarized in Table 1 and Figure 2.

**Table 1:** Baseline clinical characteristics.

Parameters	*N*
Male, *n* (%)	12 (57)
Age, years	62.8 ± 7
Vasospastic/microvascular angina, *n* (%)	11 (52)
Vasospastic angina + myocardial bridging, *n* (%)	5 (24)
Vasospastic angina + CAD, *n* (%)	3 (14,2)
MI history, *n* (%)	9 (43)
Coronary stenting, *n* (%)	9 (43)
CABG, *n* (%)	3 (14)
Myocardial bridging, *n* (%)	6 (29)
Hypertension, *n* (%)	20 (95)
Diabetes mellitus, *n* (%)	7 (33)
Stroke/TIA, *n* (%)	3 (14)
CIED, *n* (%)	3 (14)
BMI, kg/m^2^	29.4 ± 4.4
LVEF, %	61.3 ± 7.7
LAVi, mL/m^2^	39,7 ± 6.3
LVEDV, mL	116 ± 30
LVESV, mL	47 ± 21
IVS, mm	11.7 ± 1.5
LVPW, mm	10.4 ± 1.4
Total cholesterol, mmol/L	3.68 ± 1.1
LDL-C, mmol/L	1.94 ± 0.92
Triglycerides, mmol/L	1.5 ± 0.8
CKD-EPI, mL/min/1.73m^2^	84 ± 44
Hospitalization per year	3.8 per patient

MI, myocardial infarction; CABG, coronary artery bypass grafting; TIA, transient ischemic attack; CIED, cardiac implantable electronic device; BMI, body mass index; LVEF, left ventricular ejection fraction; LAVi, left atrium volume index; LVEDV, left ventricle end-diastolic volume; LVESV, left ventricle end-systolic volume; IVS, intraventricular septum; LVPW, left ventricular posterior wall.

### 3.2 Safety and procedural outcomes

**Figure 3:**
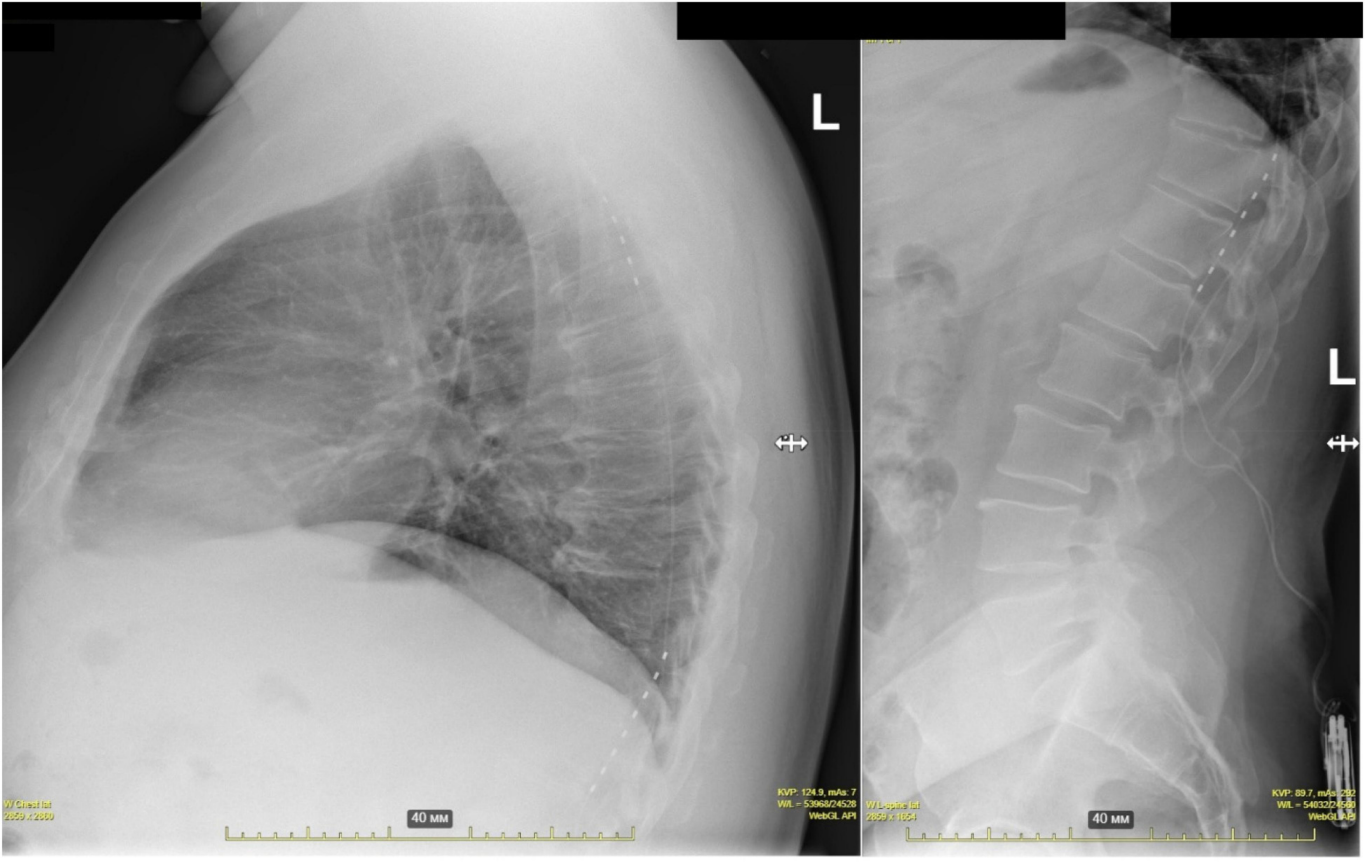
Electrode dislodgement into the lower thoracic and lumbar region.

No intraoperative complications or bleeding events occurred. Implantation of two electrodes was successful in all patients except one, where anatomical constraints limited one electrode placement. Device interrogation confirmed no interference with implanted cardiac devices in three patients, confirmed during further follow-up visits. The mean implantation procedure duration was 90 ± 32 min, with a mean fluoroscopy time of 10 ± 4.2 min. Electrode dislodgement necessitated repositioning in 19% of patients, consistent with published rates (Figure 3). Three lead dislocations occurred in patients with lumbar epidural access, and one dislocation occurred with thoracic access (*χ*^2^ = 4.5, *p* = 0.029). Device pocket infections led to extraction in 9.5% of cases. One patient developed stimulation-induced cluster-like headaches, a novel adverse event, with no structural abnormalities detected on imaging. At one-year follow-up, stimulation therapy remained active in 81% of patients.

### 3.3 Clinical and patient-reported outcomes

**Figure 4:**
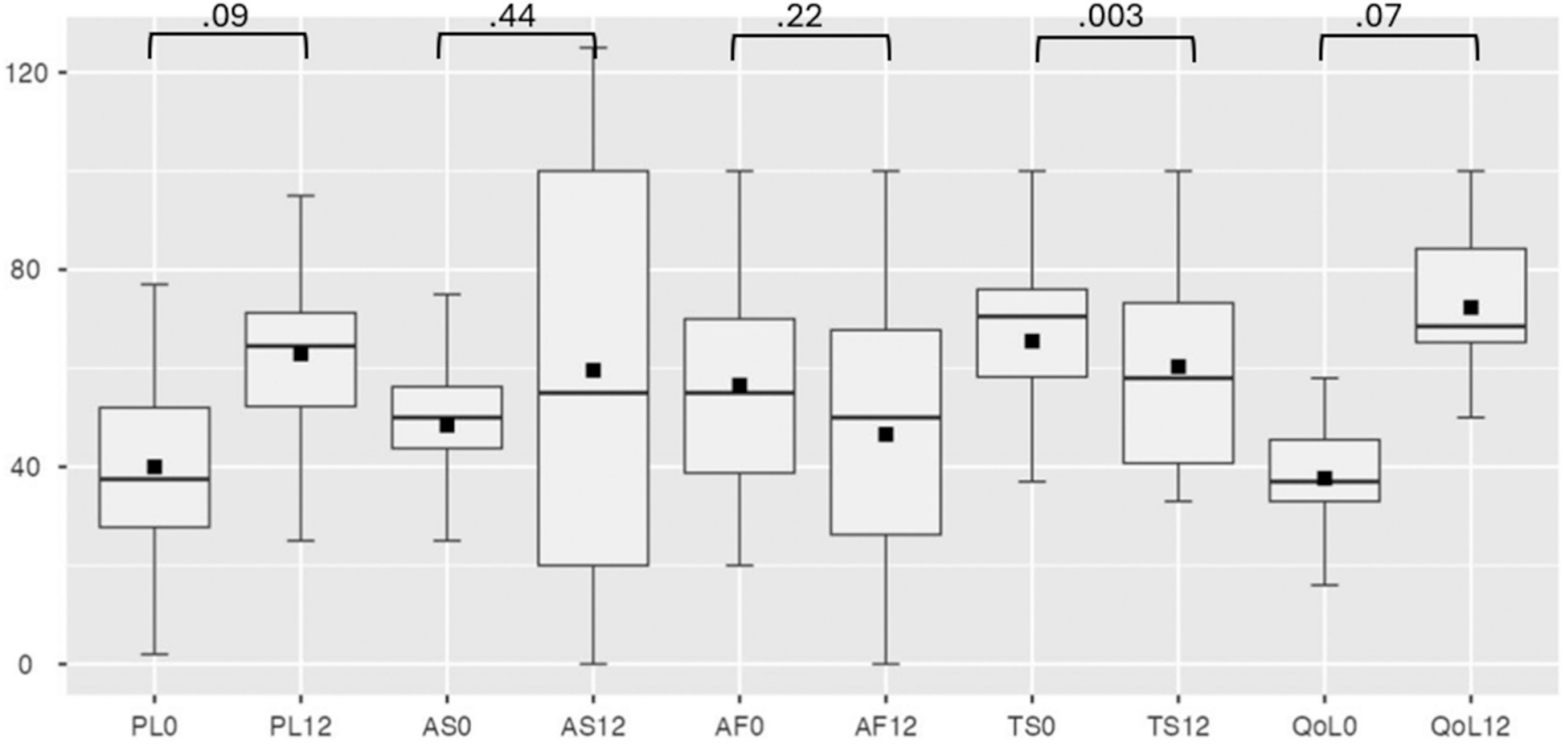
Seattle angina questionnaire at baseline and follow-up. PL, physical limitation; AS, angina stability; AF, angina frequency; TS, treatment satisfaction; QoL, quality of life, where 0 is at baseline, 12—at 12-month follow-up.

**Figure 5:**
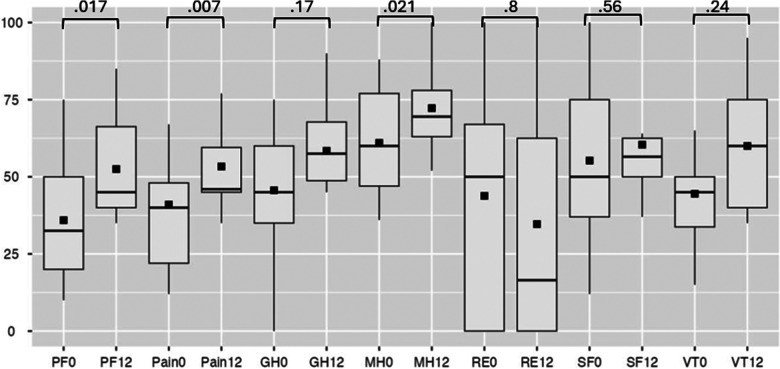
SF-36 at baseline and follow-up. PF, physical functioning; GH, general health; MH, mental health; RE, role-emotional; SF, social functioning; VT, vitality, where 0 is at baseline, 12—at 12-month follow-up.

Efficacy analysis excluded patients with device extraction. SAQ-19 scores showed non-significant improvements in four domains (Physical Limitation, Angina Frequency, Angina Stability, Quality of Life) but a decrease in Treatment Satisfaction (*p* = 0.003) (Figure 4). Secondary analysis via SF-36 indicated statistically significant improvement in physical functionating (36%–52%, OR: 17; 95% CI: 3.80–30.73, *p* = 0.017), mental health (61%–72%, OR: 21; 95% CI: 3.98–38.01, *p* = 0.021) and a trend toward reduced pain scores (41%–53%, OR: 14; 95% CI: 5.06–24.58, *p* = 0.007) (Figure 5).

### 3.4 Hospitalizations and coronary interventions

During 12 months, three patients underwent coronary stenting triggered by acute coronary syndromes or positive stress tests. Despite this, hospitalizations decreased significantly from 3.8 to 0.5 per patient per year (OR: 0.13; 95% CI: 0.09–0.2, *p* = 0.03), suggesting improved disease management and symptom control. No major adverse cardiac events (MACE) were recorded during the one-year follow-up period.

### 3.5 Nitrate therapy

**Figure 6:**
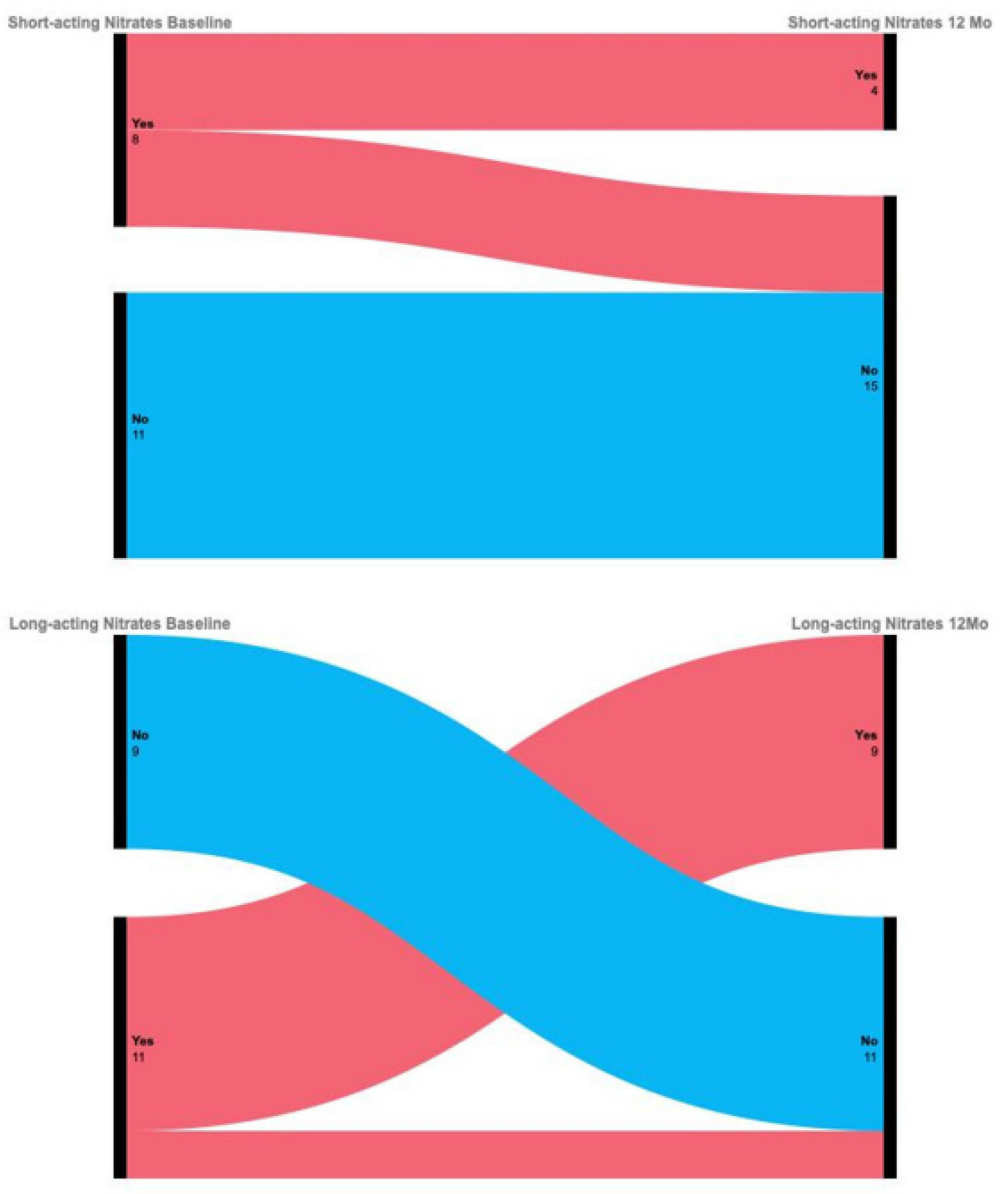
Changes in nitrate use during 12-month follow-up.

No new initiations of nitrate therapy occurred among previously nitrate-naïve patients. Additionally, two patients discontinued long-acting nitrates and four discontinued short-acting nitrates during follow-up (Figure 6), although changes did not reach statistical significance due to sample size limitations.

### 3.6 Adversarial validation

Adversarial validation demonstrated no meaningful discriminative patterns between temporal subgroups (ROC AUC = 0.52), suggesting that subsets remained consistent throughout the study period. This stability supports the appropriateness of the performed outcome analysis without temporal stratification.

## 4 Discussion

Our single-center study evaluating invasive SCS for refractory angina yielded several important insights. The study demonstrates that SCS was associated with a significant reduction in hospitalizations and marked improvements in key patient-reported outcomes, including physical functionating, pain intensity and mental health status. This positive clinical response, however, was not accompanied by a statistically significant improvement in disease-specific functional status as measured by the SAQ-19 questionnaire. Furthermore, several secondary findings of interest emerged from the data. First, the study population was clinically heterogeneous, with vasospastic and microvascular angina as the most prevalent diagnoses. Second, pharmacological management typically involved multi-drug regimens, most frequently combining beta-blockers with either dihydropyridine calcium channel blockers or long-acting nitrates. Third, although SCS demonstrated therapeutic potential, the procedure was associated with notable risks. The most common complications were lead dislodgement and device pocket infections. Dislocations were more frequently associated with lumbar access. Importantly, despite universal antiplatelet therapy related to the patients’ high cardiovascular risk, no procedural bleeding events were observed. An unexpected adverse event was the development of stimulation-induced headaches in one patient.

The prevalence of refractory angina is increasing, driven by advances in diagnostic capabilities—particularly the widespread adoption of coronary angiography—and recognition of mixed coronary pathologies such as concomitant atherosclerosis and vasospasm (13). Notably, the ORBITA-2 trial demonstrated that angina persists post-coronary stenting in approximately 60% of cases, further complicating the clinical management of these patients (14). In our cohort, among eleven patients diagnosed with vasospastic angina, five patients had myocardial bridging and three had obstructive coronary artery disease, underscoring the heterogeneity of this population and the need to explore diverse treatment modalities. Over half of the patients had undergone prior coronary interventions with suboptimal clinical response. The potential differential effects of SCS on the distinct pathophysiological entities of vasospastic angina and microvascular angina remain a subject of scientific inquiry. Our cohort included patients with both conditions, and while a formal subgroup analysis was not feasible due to diagnostic overlap and the lack of ergonovine testing, this distinction warrants theoretical consideration. We hypothesize that SCS could be particularly effective in vasospastic angina due to its direct anti-sympathetic action, which may prevent coronary spasm. In contrast, its benefit in microvascular angina likely relates more to neuromodulation and reduced pain perception. While the present study was not powered for a formal subgroup analysis, this distinction remains an important consideration for understanding SCS and should be investigated in future phenotyped cohorts.

Our findings emphasize several clinical considerations in managing refractory angina patients. Despite prior revascularization, all patients remained on antianginal therapy, with strong associations between beta-blockers and other drug classes. A Sankey diagram visualization confirmed that some patients concurrently received nitrates and ranolazine, consistent with current guideline recommendations (7).

Major trials have shown that LDL-cholesterol lowering therapies reduce MACE by 20%–45%, with the magnitude of benefit correlating with treatment intensity; maximum-dose statins and combination therapies (e.g., statins plus ezetimibe or PCSK9 inhibitors) confer the greatest cardiovascular protection (15). Nevertheless, our data revealed suboptimal lipid control, with many patients failing to reach LDL targets despite availability of PCSK9 inhibitors. This is particularly relevant given the key role of dyslipidemia in refractory angina pathogenesis. Elevated LDL cholesterol and reduced HDL levels contribute to endothelial dysfunction and impaired microcirculation, while promoting atherosclerosis progression and chronic inflammation (16). These factors exacerbate myocardial ischemia, especially where collateral circulation is limited, likely underpinning symptom persistence despite optimal antianginal regimens (17). These observations highlight the complexity of managing this high-risk group and underscore the importance of intensified lipid management and personalized antianginal therapy.

It should be noted that our study protocol did not include an initial test stimulation period with temporary electrodes prior to permanent system implantation. This decision was based on accumulating evidence suggesting that the predictive value of short-term test stimulation for long-term clinical efficacy in refractory angina patients is limited. Several studies have highlighted that transient test trials—typically lasting a few days—may not accurately capture the sustained response to spinal cord stimulation, given the complex and often delayed neuromodulatory effects involved in chronic angina management (18, 19). Moreover, the “honeymoon effect”, whereby patients initially respond favorably due to nonspecific or placebo-related factors, has been described in both pain and angina neuromodulation trials, potentially leading to overestimation of true long-term benefit during short-term testing (20). Furthermore, procedural considerations and current recommendations in cardiac SCS protocols recognize that the evaluation of true therapeutic efficacy requires prolonged observation under chronic stimulation conditions, with continuous neurostimulation and patient-managed dosing adjustments over weeks to months (21). Therefore, in our protocol, we proceeded directly to permanent implantation and evaluated clinical outcomes over extended follow-up, thus better reflecting real-world practice and allowing more accurate assessment of sustained benefit and device safety.

Reported rates of electrode dislodgement range from 8.3% to 23% even in experienced centers. Our dislodgement rate of 19% aligns with this literature (6, 22). Our analysis revealed that 75% of dislocations (3/4 cases) occurred following lumbar access procedures. Implantations were performed by two surgeons who selected the access site based on personal experience, notwithstanding protocol recommendations favoring a thoracic approach (2). Lumbar approach is often considered as a technically simpler and safer alternative, allowing one to avoid the direct risks of dural puncture in the narrow thoracic epidural space near the spinal cord, the data obtained in our and previous studies indicate the opposite. It is possible that the longer and more tortuous path of the lead from the lumbar level to the target at the Th1–Th4 segments creates an additional risk of lead migration or unstable fixation in the long term, which negates the initial safety benefit of the puncture. Thus, the apparent technical simplicity of the lumbar approach may mask other technical nuances that lead to an increased rate of postoperative complications (primarily, electrode dislodgement), as identified in our analysis (23, 24).

The pocket infection rate in our cohort (9.5%) also raises concern and warrants ongoing attention (25). A particularly noteworthy case involved a female patient who developed acute cluster-type headaches associated with neurostimulator activation. These episodes, characterized by sudden facial flushing and severe cephalalgia, occurred in a patient without prior migraine history. Radiological assessments—including lead position verification via x-ray and brain MRI—excluded electrode displacement and intracranial pathology.

While awaiting results from the SCRAP trial (26), which employs positron emission tomography-quantified myocardial perfusion as its primary endpoint, patient-reported outcomes assessed using validated questionnaires such as SAQ-19 remain the primary efficacy measures for SCS in refractory angina.

Previous studies have reported heterogeneous results, with improvements not consistently observed across all questionnaire domains. For instance, Vervaat et al. reported a reduction in angina frequency without corresponding quality of life improvements in SAQ-19 (6). Similarly, our study observed improvement in only selected domains of SF-36, while patients expressed dissatisfaction regarding treatment on SAQ-19. The lack of significant improvement in the primary endpoint (SAQ-19) must be balanced against the positive trends in the SF-36 questionnaire. This discrepancy may be attributed to the differing sensitivities of these instruments: while the SAQ-19 captures specific anginal symptoms, the SF-36 reflects broader improvements in general quality of life and functional status. We hypothesize that the mechanism of SCS action may be primarily associated with enhancing overall physical capacity and modulating pain perception, which does not always directly correlate with a reduction in anginal episode frequency as measured by a disease-specific questionnaire.

The SAQ-19 is a widely utilized and validated instrument for assessing health status in patients with angina pectoris. While its use is standard in the field, the interpretation of our results must be considered in the context of its inherent limitations. These include incomplete capture of all symptomatic dimensions, susceptibility to recall bias, and the potential for missing data and selection bias to influence the findings (27). Furthermore, the effectiveness of SCS therapy is notably patient-dependent, this may reflect limitations of the assessment tools, which are influenced by patients' active participation—such as toggling stimulation modes, maintaining battery charge, and modulating stimulation intensity. We acknowledge that patient adherence is a critical factor for long-term success.

Given the relatively small sample size and the observational single-arm design with close patient monitoring, we cannot completely exclude the influence of psychological adaptation, as well as placebo and Hawthorne effects, on the observed improvements in the SF-36 physical functionating, mental health and pain domains. However, considering the parallel reduction in hospitalization rates and decreased need for nitrates—alongside the similarity of our findings to those in studies with comparable designs—we believe these changes also reflect an overall enhancement in patient well-being and quality of life, and we tend to attribute them primarily to the independent effect of neuromodulation.

Despite these challenges, hospitalization rates and incidence of percutaneous coronary interventions decreased significantly in our cohort. We hypothesize that continuous SCS inhibits sympathetic nervous system activity and that protocolized pain management enables rapid angina relief, thereby reducing emergency visits and hospitalizations. Notably, there is no evidence in the literature that SCS masks myocardial infarction symptoms; two longitudinal studies have found no indication that SCS interferes with the diagnosis of acute myocardial infarction (28, 29).

Until randomized controlled trials are available, definitive conclusions remain elusive. There were no clinical signs of hyperparasympathetic activity (e.g., increased salivation, dyspepsia) observed. Regarding nitrate therapy, although no statistically significant change was noted, no escalation of treatment was required during follow-up. While meta-analyses report successful nitrate discontinuation post-SCS, the cautious approach of treating physicians—likely reflecting the complexity of this patient population—may have limited nitrate withdrawal in our study (30).

Despite high initial costs (approx. €16,050), the investment is offset within 16 months, primarily due to a dramatic reduction in hospitalizations. The annual number of inpatient days fell from 8.3 to 2.5 days, with 63% of patients avoiding hospitalization entirely post-implantation. Long-term data show a high therapy adherence rate of 91% after 4.5 years, with benefits persisting for up to 15 years, further supporting cost-effectiveness (5, 31). In our cohort, we observed an up to 8-fold reduction in hospitalizations and a 1-year adherence rate of 81%. However, a substantial complication rate—predominantly related to surgical access—undermines treatment satisfaction and leads to additional hospitalizations and costs. This highlights a critical need for standardizing the implantation protocol to improve patient outcomes and maximize the therapy's economic benefit.

### 4.1 Study limitations

Limitations include the single-center, observational design without randomization into control and study groups, and the small sample size, which limit causal inference and statistical power. However, our data support the potential of SCS as complementary therapy in refractory angina and provide a foundation for larger randomized trials.

## 5 Conclusion

Our prospective single-arm study demonstrated that SCS was associated with meaningful improvements in physical functionating, mental health and pain reduction as measured by the SF-36, accompanied by a significant decrease in hospitalization rates and a trend toward reduced nitrate use. However, no significant changes were observed in the primary outcome, disease-specific quality of life (SAQ-19), and the procedure was also characterized by a considerable conplication rate, at the same time consistent with previously published data.

Taken together, these findings suggest that spinal cord stimulation may offer clinical and quality-of-life benefits for carefully selected patients with refractory angina. However, given the observational design and limited sample size, larger randomized controlled multicenter trials are warranted to confirm the efficacy and long-term safety of SCS in this patient population.

## Data Availability

The raw data supporting the conclusions of this article will be made available by the authors upon reasonable request.
